# Supplementary material for: Enhanced electron dephasing in three-dimensional topological insulators
Source: Nat Commun. 2017 Jul 11;8:16071. doi: 10.1038/ncomms16071 (PMC5508222; doi:10.1038/ncomms16071)
Supplement: Supplementary Information [file ncomms16071-s1.pdf]

File name: Supplementary Information
Description: Supplementary Figures, Supplementary Table, Supplementary Notes and Supplementary References

File name: Peer Review File
Description:

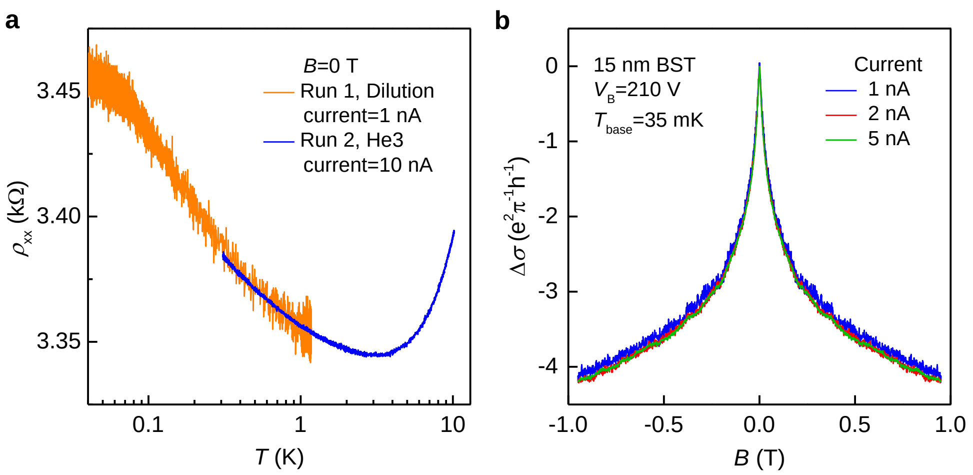

Supplementary Figure 1. Testing excitation current for electron transport measurements. (**a**) Sheet resistivity ρ_{xx} of a 15 nm $(Bi_{1-x}Sb_x)_2Te_3$ (BST) sample (Sample #1) with back-gate voltage V_B=210 V as a function of temperature in zero magnetic fields. Consistent results were obtained from two separate cool downs with different currents (1 nA and 10 nA for the measurements in a He-3 cryostat and a dilution refrigerator, respectively). (**b**) Magnetoconductivity (MC) curves of Sample #1 at V_B=210 V and T=35 mK measured with ac currents of 1 nA, 2 nA and 5 nA.

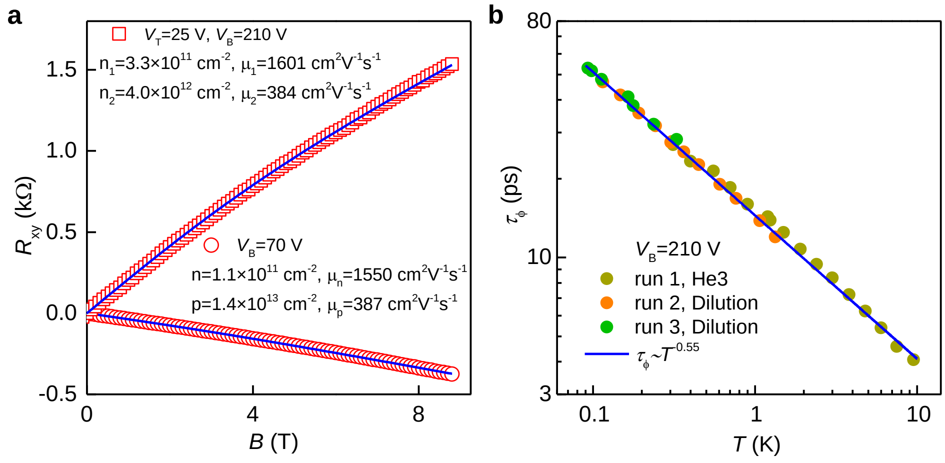

Supplementary Figure 2. Obtaining dephasing time via two-band fits. (**a**) Two-band fits of the Hall resistances of Sample #1. Only two cases with V_T=25 V and V_B=210 V (squares) and V_B=70 V and V_T=0 (circles) are shown. The sign of R_{xy} is set to positive for electron-type carriers. (**b**) Dephasing time τ_ϕ for the top surface of Sample #1 at V_B=210 V as a function of temperature (symbols). The solid line is the best fit to power law $\tau_\phi \propto T^{-p}$, which gives p=0.55.

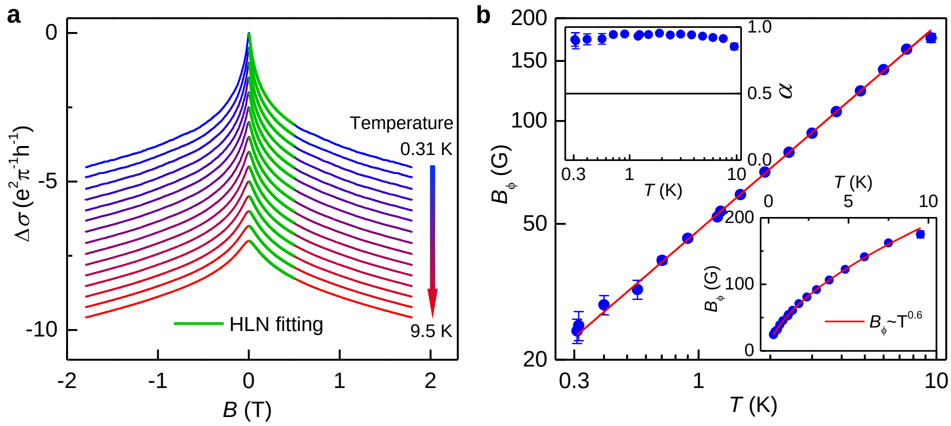

Supplementary Figure 3. Extracting dephasing fields. (**a**) MC curves of Sample #1 recorded at V_T=25V, V_B=210V in perpendicular magnetic fields and their best fits to the HLN equation [Eq. 1 in the main text] at temperatures from 0.31 K to 9.5 K. The curves are shifted vertically for clarity. (**b**) Dephasing field B_ϕ vs T. The error bars denote standard deviations of B_ϕ determined from various fitting ranges. The upper inset shows the corresponding α values, which are nearly independent of the temperature. In the lower inset, B_ϕ is plotted as a function of T in the linear scale. The red solid lines are the best fits to $B_\phi \sim T^p$, which yield p=0.6.

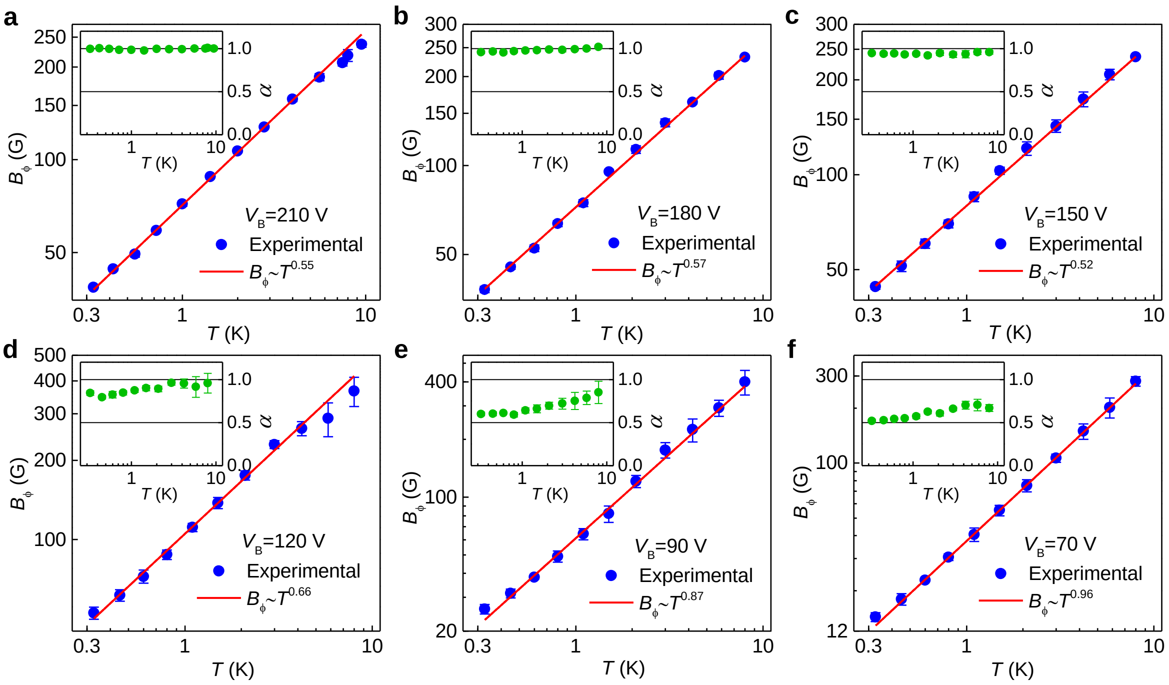

Supplementary Figure 4. Temperature dependences of α and B_ϕ at various back-gate voltages. Both were extracted from the HLN fits of the perpendicular field MCs (symbols). The red lines are the best fits to power law $B_\phi \sim T^p$. As α decreases from ~1 to ~1/2, the exponent p increases from 0.55 to 0.96.

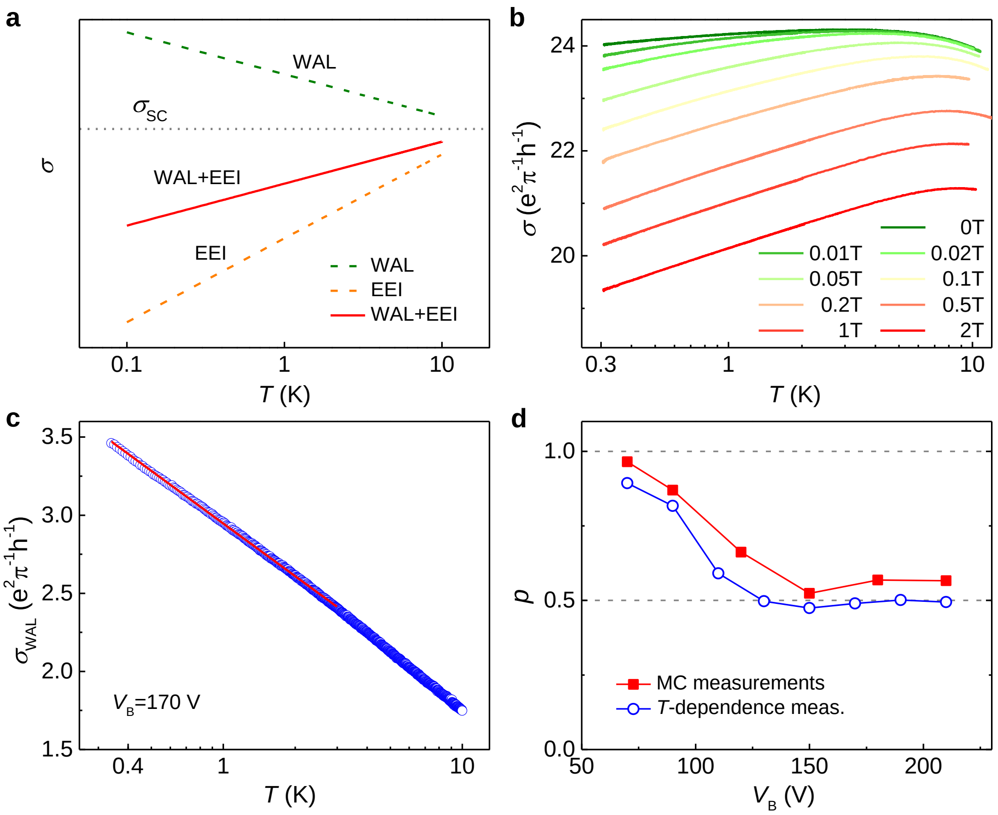

Supplementary Figure 5. Extraction of dephasing exponent *p* from the temperature dependence of conductivity. (**a**) Schematic illustration of two types of quantum correction to conductivity, the weak antilocalization (WAL) and the e-e interaction (EEI). (**b**) Conductivity of Sample #1 at back-gate voltage V_B=210 V as a function of T measured in various magnetic fields. (**c**) Conduction correction due to the WAL effect, which is obtained with $\Delta\sigma_{\mathrm{WAL}}(T) = \sigma(T)|_{B=0} - \sigma(T)|_{B=1\mathrm{T}}$ at V_B=170 V (symbols). The red line is the ln$T$ fit for obtaining $\Delta\kappa = \alpha p$. (**d**) Dephasing exponent p obtained with the T-dependent measurements (open circles) and the MC measurements (solid squares, see Fig. 4c in the main text and Supplementary Fig. 4).

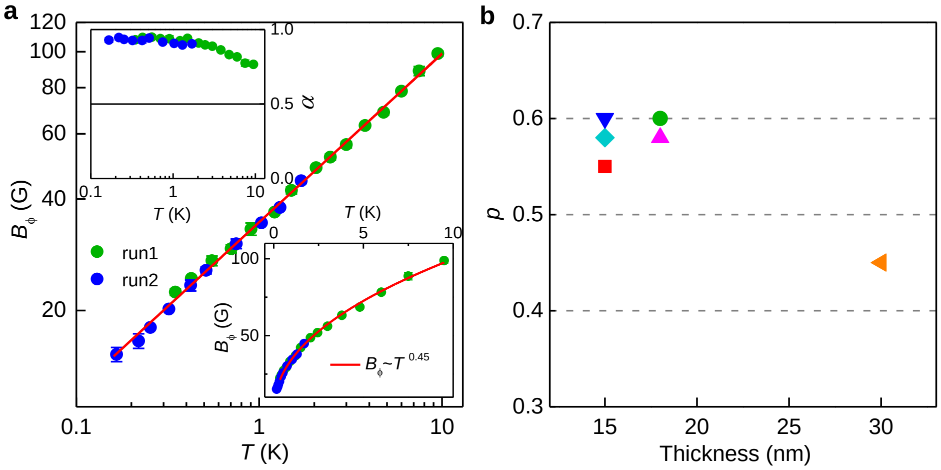

Supplementary Figure 6. Temperature dependences of dephasing field B_ϕ for several samples. (**a**) Log-log plot of B_ϕ vs T for a 30 nm BST thin film (Sample #2). The upper inset shows the corresponding α values as a function of temperature. The lower inset shows the same set of B_ϕ data but with the temperature in the linear scale. The red solid lines are the best fits to $B_\phi \sim T^p$, which yield p=0.45. (**b**) Dephasing exponent p as a function of the film thickness for Samples #1-#6 (See Supplementary Table 1 for the basic transport parameters of these samples).

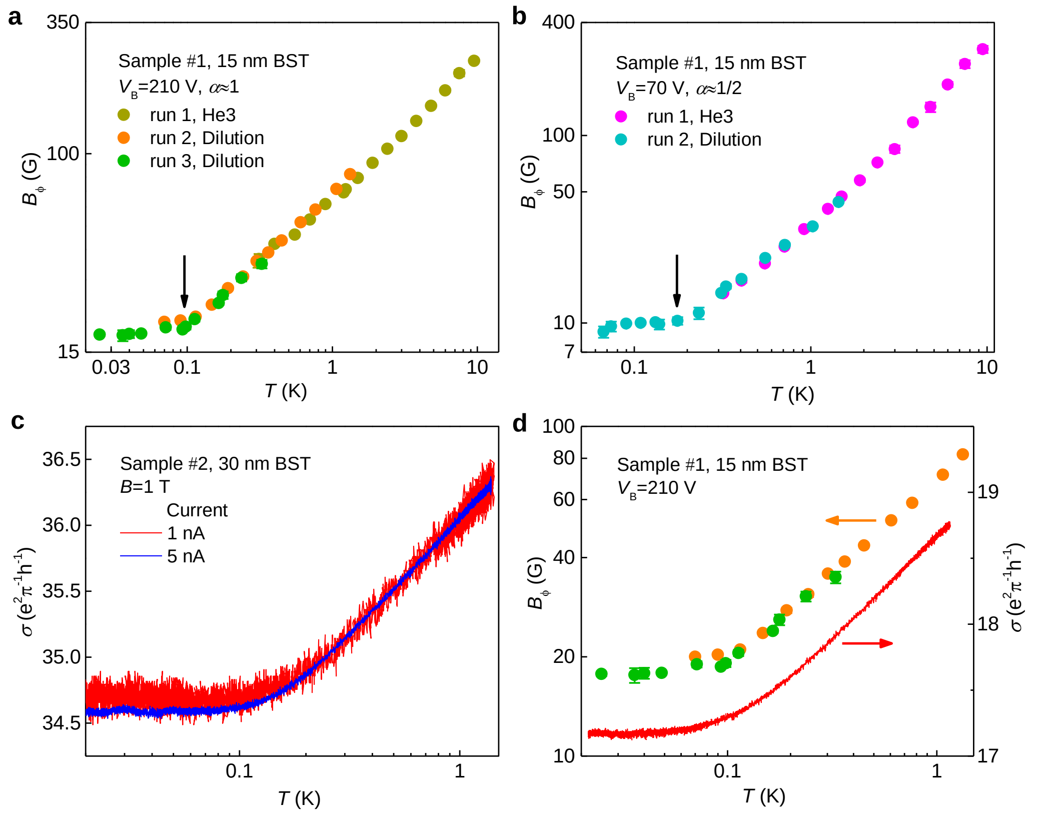

Supplementary Figure 7. Saturation of dephasing field B_ϕ at low temperatures. (**a**,**b**) B_ϕ as a function of temperature for Sample #1 in the decoupled surface-transport regime [V_B=210 V, Panel (**a**)] and in the bulk-conducting regime [V_B=70 V, Panel (**b**)]. (**c**) Temperature dependence of the conductivity of Sample #2 in the decoupled surface-transport regime measured with I=1 nA and 5 nA in a constant magnetic field (B=1 T). (**d**) T-dependences of the dephasing field and conductivity for Sample #1 at V_B=210 V.

Sample No.	Thickness (nm)	$\rho_{xx,max}$ (Ω)	V_B for $\rho_{xx,max}$ (V)	α_{max} (T=1.6 K)	V_B for α_{max} (V)
#1	15	4071	145	1.02	197
#2	30	2265	60	0.95	90
#3	18	4207	122	0.99	210
#4	18	3755	138	0.96	200
#5	15	3901	178	0.94	210
#6	15	3535	200	0.97	210

Supplementary Table 1. Basic transport parameters of highly tunable $(Bi_{1-x}Sb_x)_2Te_3$ (BST) thin films. In this work, the Sb composition in the $(Bi_{1-x}Sb_x)_2Te_3$ (BST) samples was chosen to $x > 0.9$ to prevent the Dirac point being buried in the bulk valence band. The thicknesses of the samples are in a range of 15-30 nm, which is sufficient to make the hybridization effect between the top and bottom surfaces negligible. Detailed dephasing-field measurements have been carried out on these samples, in which the chemical potential is highly tunable.

Theoretical background and methods for data analysis

Supplementary Note 1: Multi-channel quantum coherent transport

The negative magnetoconductivity (MC) due to the weak antilocalization (WAL) effect can be easily observed at low temperatures in 3D TIs (which will be referred to as TIs below). In a weakly disordered TI, the MC in low perpendicular magnetic fields usually can be described well by the Hikami-Larkin-Nagaoka (HLN) equation at the strong spin-orbit coupling limit[1] [See also Eq. 1 in the main text]:

$$\Delta\sigma(B) = -\alpha\frac{e^2}{2\pi h} f\left(\frac{B_\phi}{B}\right) = -\alpha\frac{e^2}{2\pi h}\left[\psi\left(\frac{1}{2}+\frac{B_\phi}{B}\right) - \ln\left(\frac{B_\phi}{B}\right)\right], \quad (1)$$

where $\psi(x)$ is the digamma function, and B_ϕ is the dephasing field. The prefactor α is equal to $\frac{1}{2}n_c$, where $n_c = 2\alpha$ is the number of independent transport channels with identical dephasing fields. The channel number n_c obtained from the HLN fits, however, often deviate strongly from integer values. This can be attributed to existence of more than one conduction channels. Even when the bulk conductivity is fully suppressed, both top and bottom surface states can contribute to the transport. This can be further complicated by coherent couplings between the two surfaces and between the surface and bulk.[2-5] For a coherently coupled two-channel WAL effect, the MC can be written as:

$$\Delta\sigma(B) = -\alpha\frac{e^2}{\pi h}\left[f\left(\frac{B_a}{B}\right) + f\left(\frac{B_b}{B}\right)\right], \quad (2)$$

where the two terms on the right-hand side are corresponding to two Cooperon modes, which have the same functional form as the simplified HLN equation [see Supplementary Eq. 1]. However, in many cases parameters B_a and B_b cannot be regarded as the dephasing fields. They are rather determined by a complicated function of dephasing fields $B_{\phi,1}$ and $B_{\phi,2}$, and the coupling strength between these two channels.[5,6] For a symmetric two-channel transport, i.e. $B_{\phi,1}= B_{\phi,2}= B_{\phi,0}$, the MC can be simplified into the following form:

$$\Delta\sigma(B) = -\alpha\frac{e^2}{2\pi h}\left[f\left(\frac{B_{\phi,0}}{B}\right) + f\left[\frac{B_{\phi,0}}{B}\left(1+2\frac{\tau_\phi}{\tau_s}\right)\right]\right], \quad (3)$$

where $B_s = \hbar / 4eD\tau_s$ and τ_s^{-1} is the inter-channel tunneling rate. If the inter-channel coupling is not negligible, the fit with Supplementary Eq. 1 usually yields an α value between 1/2 and 1, and the obtained B_ϕ values cannot be used to extract the dephasing rate of each surface directly. Nevertheless, there are two limiting cases that allow for straightforward evaluation of the dephasing rate in TIs. They

are delineated as follows.

One is the strong inter-channel coupling limit, i.e. $\tau_s^{-1} >> \tau_\phi^{-1}$. For a symmetric two-channel transport, the second term in Supplementary Eq. 3 can be dropped out and the transport behaves like a single channel case. The MC data can be described by the HLN equation with α=1/2, and B_ϕ is given by $B_\phi = B_{\phi,1} = B_{\phi,2} = \hbar/(4eD\tau_\phi)$, where D is the diffusion constant. This simple relation can be generalized to strongly coupled multi-channel transport with asymmetric dephasing fields (i.e. $B_{\phi,i} \neq B_{\phi,j}, i \neq j$). The MC also follows the HLN equation with α=1/2, but B_ϕ turns into a weighted average of the dephasing fields of all conduction channels, namely $B_\phi = \sum_i \left(D_i B_{\phi,i}\right) / \sum_i D_i$. If the dephasing rate of each channel has the same power-law dependence on temperature, namely $\tau_{\phi,i}^{-1} \sim T^p$. Dephasing field B_ϕ obtained from the HLN fit would also scale with T^p. Therefore, the MC data corresponding to α=1/2 provide a convenient basis for evaluating the temperature dependence of dephasing rate.

The other simple case is the decoupled symmetric two-channel regime, in which the MC can be described by the HLN equation with $\alpha = 1$. The B_ϕ value extracted from the HLN fit is same as the dephasing field of each surface, i.e. $B_\phi = B_{\phi,1} = B_{\phi,2} = \hbar/(4eD\tau_\phi)$. However, obtaining $\alpha = 1$ is a necessary but not a sufficient condition to warrant such a transport regime in TIs. The bulk conductivity must be suppressed in order to establish this so-called topological transport regime, which is ideal for pursuing quantum transport properties of the TI surface states.

Supplementary Note 2: Two-band classical transport model

The coexistence of multiple types of carriers in TIs makes it necessary to analyze the transport data with a multi-band Drude model. In the classical two-band model, the Hall resistance is given by[7]:

$$R_{xy} = \frac{B}{e} \frac{(n_1\mu_1^2 + n_2\mu_2^2) + B^2\mu_1^2\mu_2^2(n_1+n_2)}{(|n_1|\mu_1 + |n_2|\mu_2)^2 + B^2\mu_1^2\mu_2^2(n_1+n_2)^2}, \quad (4)$$

where n_i and μ_i are the carrier density and mobility for channel i, respectively. The longitudinal resistance at zero magnetic field, $\rho_{xx}|_{B=0}$, and Hall coefficient near zero field, $R_H|_{B=0}$, can be utilized

to reduce the number of free fitting parameters from four to two. Supplementary Eq. 4 can be extended to the case of more bands, but the increased number of free parameters often makes the fit less reliable. Since the most interesting case for TIs is the transport dominated by the top and bottom surfaces, we limited the quantitative analyses of the Hall effect data to the two-band fits throughout this work.

The ungated BST thin films are of p-type due to the high Sb composition. Positive gate voltages are required to deplete the carriers in the bulk. This is illustrated in Supplementary Fig. 2a with the upper Hall resistance curve, which was measured with a top gate voltage V_T=25 V and a back-gate voltage V_B=210 V. The best two-band fit yields n_1 = 3.3×10^{11} cm^{-2} and n_2=4.0×10^{12} cm^{-2}, both corresponding to electron-type carriers. Since each surface can hold up to at least 5×10^{12} cm^{-2} electrons with the Fermi level located below the conduction band minimum[8], n_1 and n_2 can be attributed to surface state carriers. It was also observed that reducing the back-gate voltage can lower n_2, and eventually the sign of n_2 reverses when V_B is sufficiently small. This suggests that n_1 and n_2 are associated with the top and the bottom surfaces, respectively. Such low carrier densities on the top surface can also exclude the possibility of topologically trivial surface states formed due to band bending[9,10].

The lower Hall resistance curve in Supplementary Fig. 2a, which was obtained with V_B=70 V and V_T=0, has negative slopes. The best two-band fit gives n_1 = 1.1×10^{11} cm^{-2} and n_2 = −1.4×10^{13} cm^{-2}, where the negative sign of n_2 suggests the hole-type carriers for the bottom surface states. Such a high hole density implies a Fermi level located below the top of the valence band, because the Dirac point is closer to the bulk valance band than the conduction band in the BST film[8]. In other words, p-type bulk carriers also participate in the transport, in addition to the carriers on the top and bottom surfaces. An accurate analysis of the Hall effect data thus requires a three-band fit, but it involves too many free parameters to be reliable. Nevertheless, the high hole density extracted from the two-band fit provides a valuable indicator for the bulk transport. It is also noticeable that the α value decreases from 1 toward 1/2 as the hole density $|n_2|$ becomes larger. This is consistent with previous work on Bi_2Se_3 thin films[2-4,8,11-14], in which the conducting bulk can couple with the surface states and lead to the reduction in the α value.

For the BST thin films studied in this work, the carrier mobility of the top surface, μ_1, is usually much larger than that of the bottom surface, μ_2, as shown in Supplementary Fig. 2a. This can be

attributed to strong interfacial scatterings at the BST/STO interface. To achieve the symmetric, decoupled surface transport, the carrier density of the bottom surface states is thus required to be higher than that of the top surface. With the carrier densities and mobilities obtained from the two-band fits, the dephasing rate of each surface can be evaluated with the following expression:

$$\tau_{\phi,i} = \frac{\hbar}{4eD_iB_\phi} = \frac{\hbar\sqrt{\pi|n_i|}}{eg_iv_FB_\phi}, \qquad (i=1,2) \tag{5}$$

where $D_i = \frac{1}{2}v_F{}^2\tau_i$ and $g_i = \sigma_i/(e^2/h)$ are the diffusion constant and the dimensionless conductivity of surface i, respectively. A Fermi velocity of $v_F \approx 4\times10^7$ cm/s[Ref.8] is used for evaluating τ_ϕ of the top surface states. As shown in Supplementary Fig. 2b, it has the same sublinear power-law dependence on temperature as the bottom surface [See Fig. 3c in the main text].

Supplementary Note 3: Electron dephasing in the variable range hopping regime

We consider Mott's theory of variable-range-hopping (VRH) transport[15], in which thermal activation assists electrons hopping between localized states at different locations. The conductivity satisfies

$$G \propto e^{-W/T}e^{-2R/\xi}. \tag{6}$$

Here the first factor on the right-hand side denotes the thermal activation over the barrier W, which is the energy difference between two localized electron states separated by R. The second factor takes account of the wave function overlapping of these two states and ξ is the localization length. The thermal activation factor favors long range hopping because W becomes smaller for larger R, whereas the tunneling factor gives advantage to short range hopping. Maximizing the conductivity described by Supplementary Eq. 6 leads to the following optimal hopping distance:

$$r \propto \left(\frac{\xi}{T}\right)^{-\frac{1}{d+1}}, \tag{7}$$

where d is the dimensionality of the electron system. Since the VRH transport is a phonon-assisted inelastic process, it can cause electron dephasing. In this regime, the dephasing length l_ϕ is believed to be set by the hopping distance r[Ref.16]. Therefore, the dephasing rate is given by:

$$\tau_\phi{}^{-1} \propto l_\phi{}^{-2} \propto T^p, \tag{8}$$

with $p=2/(d+1)$. It follows that $p=2/3$ and $1/2$ for the VRH transport in 2D and 3D electron systems,

respectively. Despite a lot of work on the VRH transport in the literature, we are not aware of any direct measurement of electron dephasing length in this strongly localized regime. Nevertheless, a couple of groups reported sublinear power-law T-dependences in a crossover regime between the diffusive and the hopping transport [17,18].

Additional transport data

Supplementary Note 4: Dephasing field extracted from the temperature dependence of conductivity

In addition to the negative MC, the WAL effect can also be manifested in the temperature dependence of conductivity. In the diffusive regime, the conductance correction due to the quantum interference leads to a ln$T$-type temperature dependence in zero magnetic fields:

$$\Delta\sigma_{WAL}(T) = -\frac{e^2}{\pi h}\alpha p \ln T, \quad (9)$$

where α is the prefactor in the HLN equation, and p is the exponent in the power-law temperature dependence of the dephasing rate (i.e. $B_\phi \propto {\tau_\phi}^{-1} \propto T^p$). As first pointed out by Altshuler and Aronov, electron-electron interaction can also give rise to a ln$T$-type conductance correction[19]:

$$\Delta\sigma_{ee}(T) = \frac{e^2}{\pi h}(1-\eta F)\ln T. \quad (10)$$

Here F is a parameter characterizing the electron screening effect, and η is a numerical factor that includes contributions from the diffusion and Cooperon channels. According to a calculation by Lu *et al.*, $\eta = 3/4$ for massless Dirac fermions[20]. If the magnetic field is not very strong, the Altshuler-Aronov correction is nearly independent of the magnetic field[19]. The measurement in a modest magnetic field (e.g. B=1 T), which is nevertheless high enough to suppress the WAL effect, can be used to evaluate the interaction effect with the κ parameter defined below:

$$\kappa_{B=1\mathrm{T}} = \frac{\pi h}{e^2}\frac{\partial\sigma(T)|_{B=1T}}{\partial lnT} = 1-\eta F. \quad (11)$$

In contrast, a similar conductivity measurement in zero fields yields

$$\kappa_{B=0} = \frac{\pi h}{e^2}\frac{\partial\sigma(T)|_{B=0}}{\partial lnT} = -\alpha p + (1-\eta F). \quad (12)$$

Therefore, the dephasing exponent p can be evaluated by a combination of the temperature-dependent measurements described above:

$$\alpha p = \Delta\kappa = \kappa_{B=1T} - \kappa_{B=0}. \quad (13)$$

Supplementary Fig. 5 shows an example with the data taken from Sample #1. The p values obtained from the temperature dependence of conductivity are in agreement with those from the MC measurements shown in the main text.

Supplementary Note 5: Data from Samples #2 - #6

In the main text, only the data taken from a 15 nm BST thin film (Sample #1) are shown. Similar results have been obtained with many other BST samples with highly tunable chemical potential. Among them, six samples were chosen for detailed transport measurements, and their basic transport parameters are listed in Supplementary Table 1. Sublinear power-law T-dependences of the dephasing rate have been observed in all of the samples in which prefactor α can be tuned to values near 1.

Supplementary Fig. 6a shows the dephasing fields for a 30 nm BST thin film (Sample #2) in the decoupled surface transport regime (i.e. α~1). The dephasing rate has a well-defined power-law dependence ($B_\phi \propto T^p$ with p= 0.45). The exponent p is smaller than 0.55 obtained for Sample #1. The p values of Samples #1-#6 are summarized in Supplementary Fig. 6b. They distribute in a range from 0.45 to 0.6, significantly lower than p=1 for the Nyquist dephasing in conventional 2D electron systems[21,22].

Supplementary Note 6: Low temperature saturation of the dephasing rate

At very low temperatures, electron-electron interaction is usually the dominant electron-dephasing source, which gives rise to a linear T-dependence of the dephasing rate in weakly disordered conductors[21-24]. As shown in the main text and above, the dephasing rate in TIs follows $\tau_\phi{}^{-1} \propto B_\phi \propto T^p$, both in the decoupled surface-transport and in the bulk-conducting regime. Supplementary Fig. 7a,b show that such a power-law dependence remains valid for a wide temperature range that spans nearly two orders of magnitude below 10 K. The dephasing rate, however, starts to saturate at a temperature between 0.1-0.2 K. Supplementary Fig. 7c shows that the saturation is not caused by the heating effects of the applied current. Since the onset temperature of the saturation is about one order of magnitude higher than the base electron temperature of our dilution refrigerator (lower than 15 mK), it cannot be attributed to electron heating from background microwaves either. Supplementary Fig. 7d shows that similar saturation behavior also appears in the temperature dependence of conductivity. During these measurements, the magnetic field was kept constant, so we could also rule out the eddy-current heating and other spurious effect related to the magnetic field ramping.

It is noteworthy that the saturation of dephasing rate has been also observed in a large variety of

low dimensional systems[21,22]. Its origin, however, has been intensively debated[23]. Both extrinsic mechanisms (e.g. magnetic impurity scattering and environmental microwaves) and intrinsic mechanisms (e.g. zero-point quantum fluctuations) have been proposed[22,23]. It is out of scope of this work to identify which mechanism can account for the low temperature dephasing saturation in TIs. Nevertheless, we speculate that it is likely related to magnetic impurities or nanometer-sized charge puddles with odd number of electrons. The issue of dephasing saturation is very important for understanding the nature of the ground states of the TI surface sates, and we will try to address it in the future work.

Supplementary References

1 Hikami, S., Larkin, A. I. & Nagaoka, Y. Spin-orbit interaction and magnetoresistance in the two-dimensional random system. *Prog. Theor. Phys.* **63**, 707–710 (1980).

2 Chen, J. *et al.* Tunable surface conductivity in Bi_2Se_3 revealed in diffusive electron transport. *Phys. Rev. B* **83,** 241304 (2011).

3 Steinberg, H., Laloe, J. B., Fatemi, V., Moodera, J. S. & Jarillo-Herrero, P. Electrically tunable surface-to-bulk coherent coupling in topological insulator thin films. *Phys. Rev. B* **84,** 233101 (2011).

4 Kim, D., Cho, Syers, P., Butch, N. P., Paglione, J. & Fuhrer, M. S. Coherent topological transport on the surface of Bi_2Se_3. *Nat. Commun.* **4,** 2040 (2013).

5 Garate, I. & Glazman, L. Weak localization and antilocalization in topological insulator thin films with coherent bulk-surface coupling. *Phys. Rev. B* **86,** 35422 (2012).

6 Raichev, O. E. & Vasilopoulos, P. Weak-localization corrections to the conductivity of double quantum wells. *J. Phys. Condens. Matter* **12,** 589 (2000).

7 Ashcroft N. W. & Mermin N. D. *Solid State Physics* (Sounders College Publishing, Fort Worth, 1976).

8 Zhang, J. *et al.* Band structure engineering in $(Bi_{1-x}Sb_x)_2Te_3$ ternary topological insulators. *Nat. Commun.* **2,** 574–579 (2011).

9 Bianchi, M. *et al.* Coexistence of the topological state and a two-dimensional electron gas on the surface of Bi_2Se_3. *Nat. Commun.* **1,** 128 (2010).

10 King, P. D. C. *et al.* Large Tunable Rashba Spin Splitting of a Two-Dimensional Electron Gas in Bi_2Se_3. *Phys. Rev. Lett.* **107,** 96802 (2011).

11 Chen J. *et al.* Gate-voltage tuning of chemical potential and weak antilocalization in Bi_2Se_3, *Phys. Rev. Lett.* **105**, 176602 (2010).

12 Checkelsky, J. G., Hor, Y. S., Cava, R. J. & Ong, N. P. Bulk band gap and surface state conduction observed in voltage-tuned crystals of the topological insulator Bi_2Se_3. *Phys. Rev. Lett.* **106,** 196801

(2011).

13 Kim, Y. S. *et al.* Thickness-dependent bulk properties and weak antilocalization effect in topological insulator Bi_2Se_3. *Phys. Rev. B* **84**, 073109 (2011).

14 Brahlek, M., Koirala, N., Salehi, M., Bansal, N. & Oh, S. Emergence of decoupled surface transport channels in bulk insulating Bi_2Se_3 thin films. *Phys. Rev. Lett.* **113**, 026801 (2014).

15 Shklovskii, B. I. & Efros, A. L. *Electron properties of doped semiconductors* (Springer Science & Business Media, 2013).

16 Ovadyahu, Z. Quantum coherent effects in Anderson insulators. *Waves Random Media* **9**, 241–253 (1999).

17 Minkov, G. M., Germanenko, A. V. & Gornyi I. V., Magnetoresistance and dephasing in a two-dimensional electron gas at intermediate conductances. *Phys. Rev. B* **70**, 245423 (2004).

18 Niimi, Y. *et al.* Quantum coherence at low temperatures in mesoscopic systems: Effect of disorder. *Phys. Rev. B* **81**, 245306 (2010).

19 Altshuler, B. L. & Aronov, A. G., Electron-electron interactions in disordered conductors. *Electron-electron interactions in disordered systems,* edited by Efros. A. L. & Pollak M. (North-Holland, Amsterdam, 1985).

20 Lu, H.-Z. & Shen, S.-Q. Finite-temperature conductivity and magnetoconductivity of topological insulators. *Phys. Rev. Lett.* **112,** 146601 (2014).

21 Imry, Y. *Introduction to Mesoscopic Physics* (Oxford University Press, Oxford 1997).

22 Lin, J. J. & Bird, J. P. Recent experimental studies of electron dephasing in metal and semiconductor mesoscopic structures. *J. Phys. Condens. Matter* **14,** R501–R596 (2002).

23 Saminadayar, L., Mohanty, P., Webb, R. A., Degiovanni, P. & Bäuerle, C. Electron coherence at low temperatures: The role of magnetic impurities. *Physica E* **40**, 12–24 (2007).

24 Mohanty, P. Of Decoherent Electrons and Disordered Conductors. *Complexity from Microscopic to Macroscopic Scales: Coherence and Large Deviations,* edited by Skjeltorp, A. T. & Vicsek, T. (Springer, Netherlands, 2002).
